# Combined action observation and motor imagery improves learning of activities of daily living in children with Developmental Coordination Disorder

**DOI:** 10.1371/journal.pone.0284086

**Published:** 2023-05-23

**Authors:** Matthew W. Scott, Greg Wood, Paul S. Holmes, Ben Marshall, Jacqueline Williams, David J. Wright

**Affiliations:** 1 Department of Psychology, Faculty of Health and Education, Manchester Metropolitan University, Manchester, United Kingdom; 2 School of Kinesiology, University of British Columbia, Vancouver, Canada; 3 Department of Sport and Exercise Sciences, Faculty of Science and Engineering, Manchester Metropolitan University, Manchester, United Kingdom; 4 Institute for Health and Sport, College of Sport and Exercise Science, Victoria University, Melbourne, Victoria, Australia; Universitat de les Illes Balears, SPAIN

## Abstract

Developmental coordination disorder (DCD) is characterised by poor motor coordination, which interferes with the ability to execute activities of daily living (ADLs). Combined action observation and motor imagery (AOMI) involves observing movement videos whilst imagining simultaneously the sensations of executing the same movement. Laboratory-based research indicates that AOMI can help improve movement coordination in children with DCD, but no previous research had investigated the efficacy of AOMI interventions for learning ADLs. This study investigated the efficacy of a home-based, parent-led, AOMI intervention for learning ADLs in children with DCD. Children with confirmed (n = 23) or suspected (n = 5) DCD (total sample n = 28), aged 7–12 years, were assigned to either an AOMI intervention or a control intervention (both n = 14). Participants attempted the following ADLs at pre-test (week 1), post-test (week 4), and retention test (week 6): shoelace tying, cutlery use, shirt buttoning, and cup stacking. Task completion times and movement techniques were recorded. The AOMI intervention produced significantly faster task completion times than the control intervention at post-test for shoelace tying, and significantly improved movement techniques for shoelace tying and cup stacking. Importantly, for children who could not tie shoelaces at pre-test (n = 9 per group), 89% of those following the AOMI intervention learnt the skill successfully by the end of the study, compared to only 44% of those following the control intervention. The findings indicate that home-based, parent-led, AOMI interventions can aid the learning of complex ADLs in children with DCD, and may be particularly effective for facilitating the learning of motor skills that do not currently exist within these children’s motor repertoire.

## Introduction

Developmental coordinating disorder (DCD) is a lifelong neurodevelopmental condition prevalent in 5–6% of children [1]. Key indicators for the presence of DCD include a delay in reaching motor milestones, a reduced motor proficiency compared to other children of their age, and importantly, movement difficulties which disrupt execution of activities of daily living (ADLs; [1, 2]). Common ADLs that children with DCD reportedly find troublesome include dressing, eating with utensils and self-care activities (e.g., brushing teeth and brushing hair; [3, 4]). Despite the acknowledgement of these difficulties, research aiming to improve the ability of children with DCD to execute fundamental ADLs is relatively sparse. Nonetheless, it has been proposed that ecologically valid task orientated approaches could facilitate the success of training ADLs in children with DCD [5, 6].

Conventional treatments for DCD often emphasise repetitive physical practice of tasks or components of these tasks [2]. It has been suggested, however, that training programs consisting of physical practice alone may not be sufficient to promote learning in children with DCD, and that mental training strategies targeting specific brain regions identified to be hypoactive in those with DCD may further improve the learning process for this population [7]. Two commonly used mental training techniques shown to be effective in children with DCD are action observation (AO) and motor imagery (MI; see [8] for review). Both AO and MI activate motor brain regions that align closely with those commonly reported to be hypoactive in individuals with DCD [8–10]. AO is an externally driven process, involving the structured and deliberate observation of human movement [11]. MI on the other hand is an internally (i.e., cognitively) driven process, involving the generation, maintenance and transformation of visual and kinaesthetic perceptual representations of movement [12]. AO and MI are referred to as forms of motor simulation; as similar motor regions are activated to movement execution during their use [13, 14]. Evidence over the past decade suggests training protocols involving the use of these motor simulations can improve movement outcomes for children with DCD. For example, AO-based training has been shown to facilitate throwing and catching accuracy and technique in children with DCD [15–17]. MI can also improve motor execution for this population across a range of tasks; for example, jumping a rope, hitting a ball with a bat and throwing and catching tasks [18, 19].

It has been suggested that these mental training strategies may enhance movement outcomes through refining internal models [20]. A prominent explanation for behavioural deficits in children with DCD is the internal modelling deficit hypothesis (IMD; [21]). Internal models–comprising the inverse and forward models–are neural representations of the environment which allow calculated movements to be performed based on perceptual feedback [22]. The forward model serves to estimate the sensory consequences of movement allowing the online correction of movement in the presence of perturbation, facilitating sensorimotor learning [23]. The premise of the IMD is that internal models are impaired in children with DCD [21], and there is now considerable evidence supporting this hypothesis [24, 25]. It has also been proposed that simulation-based training may alleviate internal modelling deficits in children with DCD [26, 27].

Recently it has been proposed that the simultaneous use of AO and MI (i.e., combined action observation and motor imagery; AOMI) could further enhance motor function for children with DCD compared to either technique alone [8, 28]. AOMI involves observing a video or live demonstration of a movement while simultaneously generating, maintaining and transforming a time-synched kinaesthetic representation of performing the same movement [29]. To date, benefits for AOMI training have been shown across both healthy and clinical populations in both laboratory-based [30–32] and home-based interventions [33]. In children with DCD, there is preliminary evidence that AOMI can enhance the imitation of simple rhythmical actions [34, 35] and improve performance of a novel computer-based visuo-motor task [36]. In the two studies by Scott et al. [34, 35], AOMI enhanced the imitative ability of children with DCD more so than AO and MI independently. Although the ability to imitate actions is reportedly impaired in children with DCD [10], these studies suggest AOMI can improve imitation ability in this population. Marshall et al. [36] found AOMI to significantly improve the performance of a virtual radial Fitts’ task in children with DCD. This computerised task required children to draw lines with a stylus on a touch-screen monitor recipoically from a central starting target to six targets presented sequentially in an arc from left to right. However, the stylus movements had a 90° counter-clockwise visual feedback rotation (i.e., upward movements causing rightward cursor movements and rightward movements causing downward cursor movements). The AOMI group completed the task significantly faster than a control group. Furthermore, the AOMI group showed more feedforward (i.e., expert-like) eye gaze strategies. The authors interpreted these eye gaze adaptations to be a marker of improvements in the forward model.

While this evidence showing benefits of AOMI training in children with DCD is encouraging, to date research has focused on abstract motor tasks and only examined acute training effects within a single testing session. No research has yet investigated the potential benefits of AOMI on activities that are essential for daily living, nor explored the longitudinal effect of AOMI training on motor learning in children with DCD. The current study, therefore, investigated the efficacy of a home-based and parent-led AOMI intervention involving the training of four ADLs which children with DCD are known to struggle executing; shoelace tying, shirt buttoning, cutlery use and cup stacking [3, 37]. Based on previous literature [35, 36] it was hypothesised that AOMI would improve the acquisition and retention of the four ADLs, evidenced by significantly better performance across all tasks in the AOMI group at post-test and retention phases, compared to the control group.

## Materials and methods

### Participants

A power analysis was initially conducted using the repeated measures, within-between interaction test function in G*Power 3.1. Estimates were based on previous motor learning research by Marshall et al. [36] where children with DCD learned a novel visuomotor task via an AOMI intervention similar to that used in the current study. According to the effect sizes for a group by time interaction (*fs* = 0.47) and α set at 0.05, 6 participants would be required per group (12 participants total) to detect significant behavioural differences with a power of 90%. Subsequent recruitment was conducted to ensure analyses were adequately powered and to accommodate any potential dropout across the longitudinal intervention.

Twenty-eight children aged 7 to 12 years (21 male, 7 female; age M = 9.57, SD = 1.39) with confirmed (n = 23) or suspected (n = 5) DCD were recruited from England, UK (see Table 1 for group demographics and characteristics). Following a similar recruitment procedure to Marshall et al. [36], participants were recruited through online support groups for parents/guardians of children with DCD. Those who volunteered then completed the Developmental Coordination Disorder Questionnaire (DCDQ; [38]) to provide an indication of their child’s movement capabilities as a screening tool for eligibility. Families were invited to the university if the responses indicated that their children scored within the criteria for potential DCD on the DCDQ (i.e., scores between 15–57, second to third quartile), and if parent reports indicated that the children did not have any co-occurring medical condition known to further impair learning or motor function (e.g., cerebral palsy, hemiplegia or muscular dystrophy), learning difficulties, or attention deficit hyperactivity disorder. Controlling for learning difficulties and attention deficit hyperactivity disorder in particular were attempts to ensure that the children who took part had the capacity to engage in the AOMI intervention. Invited children completed the Movement Assessment Battery for Children-2 (MABC-2; [39]). Children who had an official diagnosis (i.e., diagnosed by a therapist as meeting the Diagnostic and Statistical Manual of Mental Disorders, 5th edition, criteria) and also scored below the 5^th^ percentile on the MABC-2 were classified as confirmed DCD and those without a therapist-confirmed DCD diagnosis but who scored below the 5^th^ percentile were classified as suspected DCD. Ethical approval was granted by the Health and Education Research Ethics and Governance Committee at Manchester Metropolitan University (Approval reference number: 17782), and both parents/guardians and children provided their written informed consent and assent to participate, respectively. In addition to the final sample of 28 participants, four participants initially recruited and enrolled to the intervention (2 AOMI, 2 Control participants) were excluded due to their non-completion of the study (insufficient number of training sessions completed during the training phase for three participants [< 75% of training sessions] and one non-completion of the retention phase).

**Table 1:** Group demographics including movement assessment battery for children 2^nd^ edition (MABC-2) scores and movement imagery questionnaire for children (MIQ-C) scores. *—Characteristics included in the minimization procedure for group allocation.

Group	N	Age* (M ± SD)	Sex*	Diagnosis	MABC-2 manual dexterity standard score* (Median, interquartile range)	MABC-2 overall standard score (Median, interquartile range)	MIQ-C Internal visual imagery (Median, interquartile range)	MIQ-C External visual imagery (Median, interquartile range)	MIQ-C Kinaesthetic imagery* (Median, interquartile range)
AOMI	14	9.50 ± 1.34	11 male, 3 female	12 confirmed, 2 suspected	3.5, 2.25	3, 2.25	5.5, 1.13	5.75, 1.56	5.5, 1.13
Control	14	9.64 ± 1.39	10 male, 4 female	11 confirmed, 3 suspected	3, 2	3.5, 3	5.25, 1.5	5, 2.06	5.25, 1.5

### Design

This study used a mixed measures design, in which two groups (experimental and control) completed pre-test, post-test and retention test measurements to determine the efficacy of a home-based AOMI intervention for children with DCD. Interventions were delivered at the participants’ home by parents after receiving training in their respective intervention. After the pre-test, children were randomly allocated to either an AOMI group or a control group (both n = 14) via minimisation [40], a recognised and acceptable randomisation procedure [41]. This procedure minimised between-group differences in the following factors after completion of the pre-test: age, sex, manual dexterity scores (MABC-2 component), pre-test performance times (averaged across completed performances), number of ADLs successfully completed (see results sections for summary) and kinaesthetic imagery ability (Movement Imagery Questionnaire for Children component; MIQ-C [42]). Immediately after the pre-test measurements were completed, the primary researcher entered these values into the minimisation spreadsheet (accessed at [40]), which generated the group allocation. An independent samples t-tests confirmed there to be no age differences between groups and independent samples Mann-Whitney U tests confirmed no group differences for MABC-2 and MIQ-3 scores (all *p*s >.05; see Table 1). Parents and children were blinded to the nature of the study research question and to which intervention was the intervention of interest.

#### Activities of daily living

**Fig 1:**
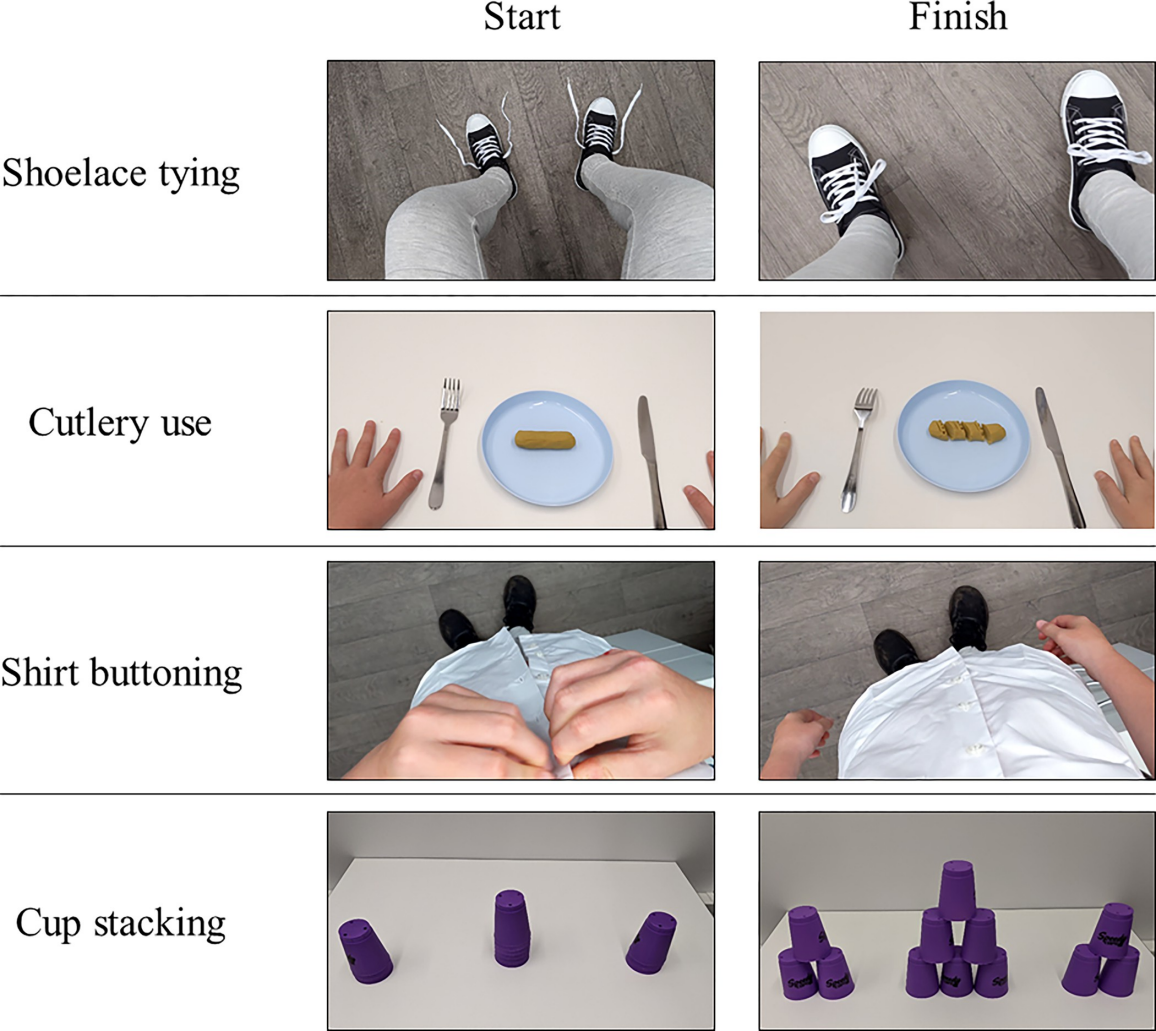
Activities of daily living. Pictorial illustration of each ADL and their respective start and finish. Screenshots were taken from the videos developed for the AOMI intervention.

The ADLs selected for the current study were shoelace tying, cutlery use, shirt buttoning and a cup stacking task (see Fig 1). The choice of ADLs were informed by recent research by Licari and Williams [3] where 71% and 63% of parents of children with DCD reported dressing and eating with utensils, respectively, to be particularly difficult ADLs. Each ADL had a clearly defined start and completion point (see Fig 1). Participants attempted to learn shoelace tying using the ‘single loop’ knot technique (see [43] for a detailed description of the technique). One trial completion for this task was defined as both shoes being tied. For cutlery use, participants used a knife and fork to cut a sausage shaped piece of play-doh into 4 roughly equal-sized pieces using 3 cuts. The shirt buttoning task involved wearing and buttoning a school shirt, with 5 correct button fastens classed as a completion for this task. The cup stacking task involved a medium difficulty formation often referred to as a ‘3-6-3’ arrangement (see [44]), recently used to assess AOMI training in a previous study [45]. This required participants to create a central pyramid-shaped stack of 6 cups flanked by a smaller pyramid stack of 3 cups on each side (i.e., 3-6-3). Children were required to only “up stack” the cups and not to complete the “down stack” phase typically performed in competitions (see Fig 1). Although cup stacking in this manner is not strictly an ADL, this bimanual coordination task involves movements which may translate to common ADLs (e.g., stacking items on a shelf), and provided an enjoyable and motivating component for the children.

### Procedure

#### Pre-test

Participants first completed the full age-appropriate version of the MABC-2, involving the measurement of manual dexterity ability, catching and throwing, and balance ability. Following this, children completed the MIQ-C [42] to assess movement imagery ability across three subscales; internal visual, external visual and kinaesthetic imagery. Children then attempted to complete 5 trials for each of the four ADLs. Five total trial attempts were selected based on pilot testing to prevent fatigue or boredom associated with more task repetitions. To further help negate these potential negative training effects, children were offered breaks throughout the session. Children were encouraged to perform the tasks in the following order: 1) cup stacking, 2) cutlery use, 3) shirt buttoning, and 4) shoelace tying. However, flexibility in the order of tasks was permitted to maintain participant interest and allow for data collection to be completed. Before attempting the block of five trials for each ADL, children were shown two videos of the tasks being performed with a conventional strategy. Children were then instructed to attempt to copy the technique shown as quickly and accurately as possible (see [36] for similar instructions). Children self-initiated each trial by pressing a push-button timer prior to beginning the task and then pressing the button again upon task completion.

#### Training phase

**Fig 2:**
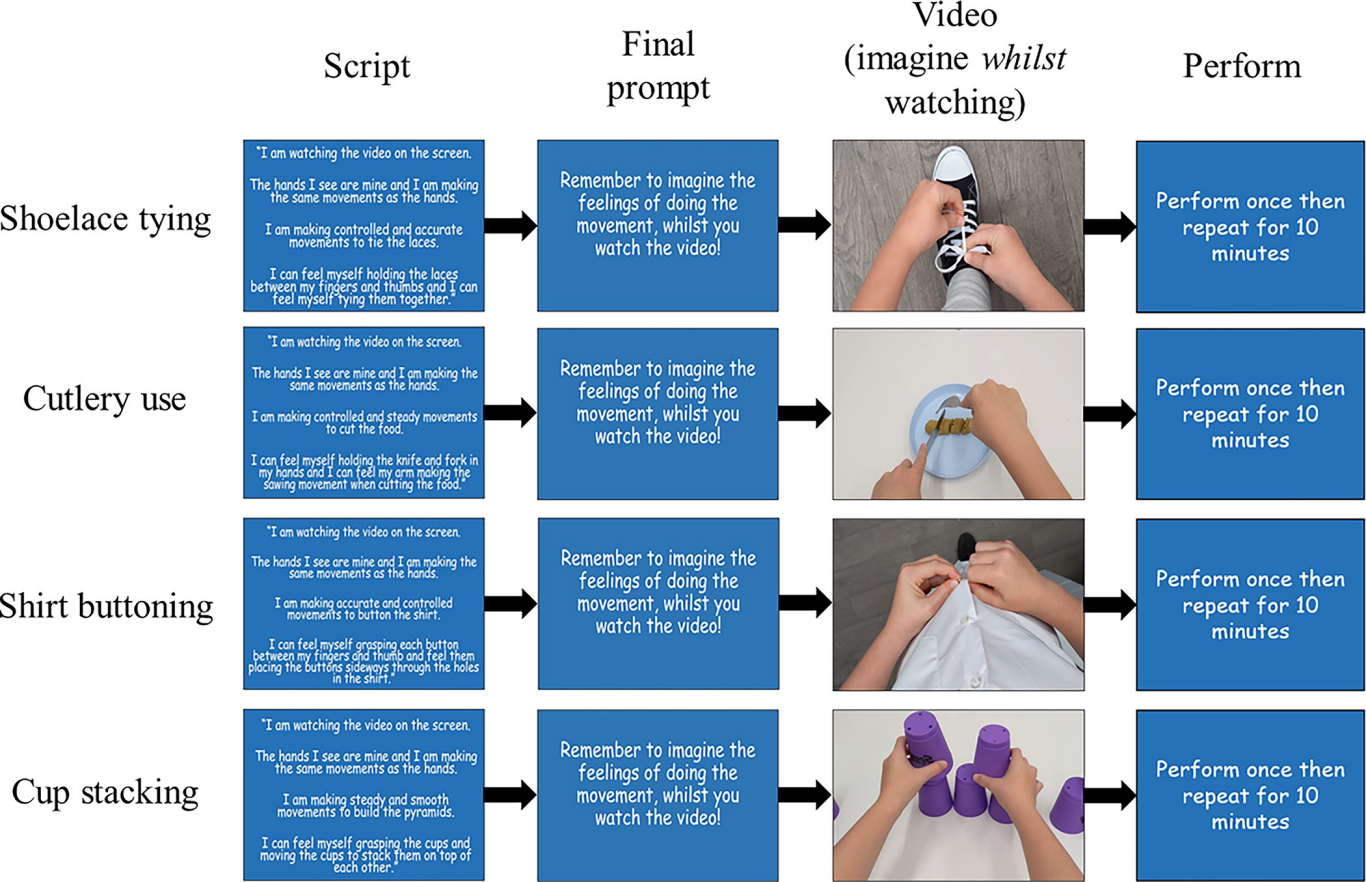
Simplified training structure for each ADL for the AOMI intervention. Children were instructed to read the scripts, imagine the content in time with the video of the task and then perform the action once. This process of reading, imagining whilst watching and then performing was repeated for 10-minutes for each ADL.

*AOMI group*. Children and parents in the AOMI group were informed that the aim of the study was to investigate whether the implementation of AOMI alongside physical practice may facilitate the learning of ADLs. Accordingly, both parents and their children were introduced to the concept of AOMI, and the parents were trained on how to deliver an AOMI intervention at home using a tablet computer (10.1-inch Samsung Tab A) with the AOMI intervention uploaded (see Fig 2). Participants were instructed to complete four 40-minute training sessions per week for four weeks. In each training session they were asked to spend 10 minutes practicing each ADL, adhering to the repetitive structure of one AOMI trial followed by one physical practice trial, before moving on to the next ADL. Participants were free to self-select the order in which they practiced the four ADLs. In total, participants completed two hours and forty minutes training each ADL, totalling 10 hours and forty minutes of training across the whole intervention. The intervention involved children first reading an imagery script tailored to the task they were training. These scripts were written in the first person and encouraged the child to consider the hands they would watch as their own and that they could feel the movements and the items used for the task (see [36]). After the script, participants saw a final prompt reminding them to imagine the feeling of performing the movement *during* the video which would play next. Participants then saw a video of a proficient child model performing the same ADLs attempted at pre-test. During the video, children were instructed to imagine, simultaneously, the feelings and sensations associated with executing the action and interacting with the items. Following an AOMI trial, on-screen instructions then prompted the child to physically execute one repetition of the ADL (see Fig 2).

Videos were developed incorporating various PETTLEP principles into the AOMI intervention [29, 46] and were presented in real-time and from a first-person visual perspective (see Fig 2). These manipulations optimised perspective and angle for learning the manual dexterity tasks [8]. To ensure anatomical and biomechanical similarity, the model was a proficient child (typically developing 13-year-old male) and videos were mirrored horizontally for both left- and right-hand dominant individuals to facilitate imitation (e.g., [35, 47]). Children received a pack containing shoes, cutlery set, shirt and stacking cups, identical to those used at pre-test and depicted in the videos and were instructed to use them when completing the AOMI and physical practice trials; a factor considered to enhance the efficacy of imagery interventions [46, 48].

*Control group*. Children and parents in the control group were informed that the aim of study was to investigate whether the integration of computer games alongside physical practice may facilitate the learning of ADLs by ‘gamifying’ training to alleviate boredom associated with repetitive task practice [49], and promoting a distributed, rather than massed, practice schedule [50]. Tablets were provided to families with a game called Cut the Rope downloaded to use for the duration of the study. This game was selected as playing it required an element of fine motor planning and execution but was unrelated to the ADLs being practiced. Following a similar structure to the AOMI group, participants were instructed to complete four 40-minute training sessions per week for four weeks. In each training session they were asked to spend 10 minutes practicing each ADL, adhering to repetitive structure of one trial of Cut the Rope followed by one physical practice trial, before moving on to the next ADL. This ensured a comparable amount of physical practice and interaction with the tablet between the two groups. In addition, those in the control group received the same training items for use during the physical practice trials (i.e., the tablet, shoes, shirt, cutlery set and stacking cups) as the AOMI group. Collectively, the provision of these activities, instructions and equipment ensured that the only difference experienced between the two groups throughout the training period related to the primary variable of interest (i.e., the AOMI intervention vs the computer game control task). This represents a more rigorous and stringent control to determine the potential benefits of AOMI than would a ‘do nothing’ control group common in motor learning research (e.g., [19, 51]).

*Adherence and monitoring*. To ensure adherence throughout the intervention, participants in both groups maintained contact with the research team via weekly video calls and email. This provided ample opportunities to discuss progress and answer questions about their respective interventions. Furthermore, both children and parents received a booklet tailored to their respective intervention. The children’s booklets, titled ‘The Young Scientist’s Diary’, gave a brief and simplified overview of their intervention. Children were asked to collect data as the young scientist by reporting how much they enjoyed each training session, using a Likert-type scale developed using emojis (see Table 1 in S1 File). Quantifying enjoyment also reinforced the narrative provided to the control group (i.e., using games to alleviate practice induced boredom), encouraging adherence. The adult booklets, on the other hand, contained more detailed information on their intervention and a diary for monitoring the time and date of sessions, task completions and their perception of their child’s motivation for the sessions (see Table 2 in S1 File). Both child and parent booklets included optional text boxes allowing children and parents to provide valuable feedback on their intervention. The use of such booklets has been incorporated previously into imagery-based interventions (see [18]) and home-based interventions [52] for children with DCD with positive outcomes.

#### Post-test

After a 4-week home-based intervention, participants returned to the university for post-test measurements. Participants first completed the MIQ-C and the manual dexterity component of the MABC-2 to assess whether any improvements in ADLs transferred to these tasks. Participants then attempted each ADL five times with the same performance measures recorded as at pre-test. Children also completed a questionnaire evaluating their intervention, providing an indication of how beneficial they believed it to be and which aspects they felt were helpful. This form also allowed parents to share their views on the intervention. Upon completion of the post-test, participants returned their tablets and training equipment to the research team to ensure training could not be conducted during the retention period.

#### Retention

Participants returned to the university for a retention test two weeks after the post-test. For the duration of this 2-week retention period participants were asked to cease specific training of the ADLs but to continue with their typical weekly routines. The retention phase involved replicating the post-test protocol, minus the evaluation questionnaire. Upon completion of this session all participants received a £25 gift voucher as a thank you for participation and had their tablet and training equipment returned to them to allow them to continue with the training should they wish. Those participants who had been in the control group also received the AOMI intervention software uploaded onto their tablet and were educated in its delivery to allow them the opportunity to experience the intervention. All pre, post and retention tests were conducted by the primary researcher.

### Measures

#### Performance times

On each visit children completed 5 trials of each ADL while being timed (secs). Participants self-initiated each trial by starting the timer and then stopped the timer after finishing each trial. If children were unable to complete one trial of an ADL, incompletion times were imputed to allow analysis of these trials (see [53, 54] for similar procedures). Incomplete times were calculated as the mean of all participants’ pre-test performances for that task plus two and a half standard deviations (incompletion time = pre-test M + 2.5*SD).

#### Technique ratings

Technique rating scales were developed ranging from 0–5 (see S2 File), these were tailored to each task to quantify strategies used during the tasks and to assess the child’s movement quality (e.g., [15, 17]). For each task 0 represented an incompletion and 5 represented use of the same technique demonstrated in the videos shown prior to attempts which children were asked to copy. To assess inter-rater reliability, the primary researcher and one other member of the research team rated 10% of trials which were randomly selected. This produced an inter-rater reliability score of 81%, indicating a strong level of agreement [55]. Accordingly, the remaining 90% were scored solely by the primary researcher.

### Data analysis

Data sets were analysed using mixed effects models. Mixed effects models, while similar to typical mixed measures ANOVAs, allow the inclusion of all within-participant observations without aggregation or violating assumptions of independence through accounting for random effects [56]. Furthermore, by introducing random effects, mixed effects models can account for individual responses (i.e., inclusion of random intercepts and slopes for participants). Separate mixed effects models were used to assess dependent variables for each ADL (i.e., performance times and technique ratings) as a function of fixed effects (group, time, and their interaction) and random effects (subject). Analyses were conducted and reported following similar protocols to recent simulation-based motor learning research [57, 58]. Performance time data were analysed using linear mixed effects (LME) models in R [59], using the lme4 package. The ordinal technique rating data was analysed using cumulative link mixed models using the ordinal package, incorporating the same mixed and random effect as the LME models. Likelihood ratio tests were conducted to determine the best model fit for data sets using Akaike Information Criterion [60]. The addition of both random intercepts and slopes for subjects significantly improved the fit of the models and were included. Where appropriate and adequately powered, exploratory analyses were conducted on subgroups of participants who were unable to complete certain ADLs at pre-test, to determine true motor learning effects of the interventions. Due to the reduction of data for these analyses (i.e., the exclusion of data for those who were able to complete the tasks at pre-test), the previous models were too complex, and therefore, mixed measures ANOVAs were used for these analyses. Differences across time were followed up with Tukey’s HSD *post-hoc* tests using the multicomp package in R [61].

## Results

For efficiency, reporting of findings was limited to those appropriate to the outlined hypotheses. Complete results from LME models for performance times can be found in S3 File.

### Shoelace tying

#### Performance times

**Fig 3:**
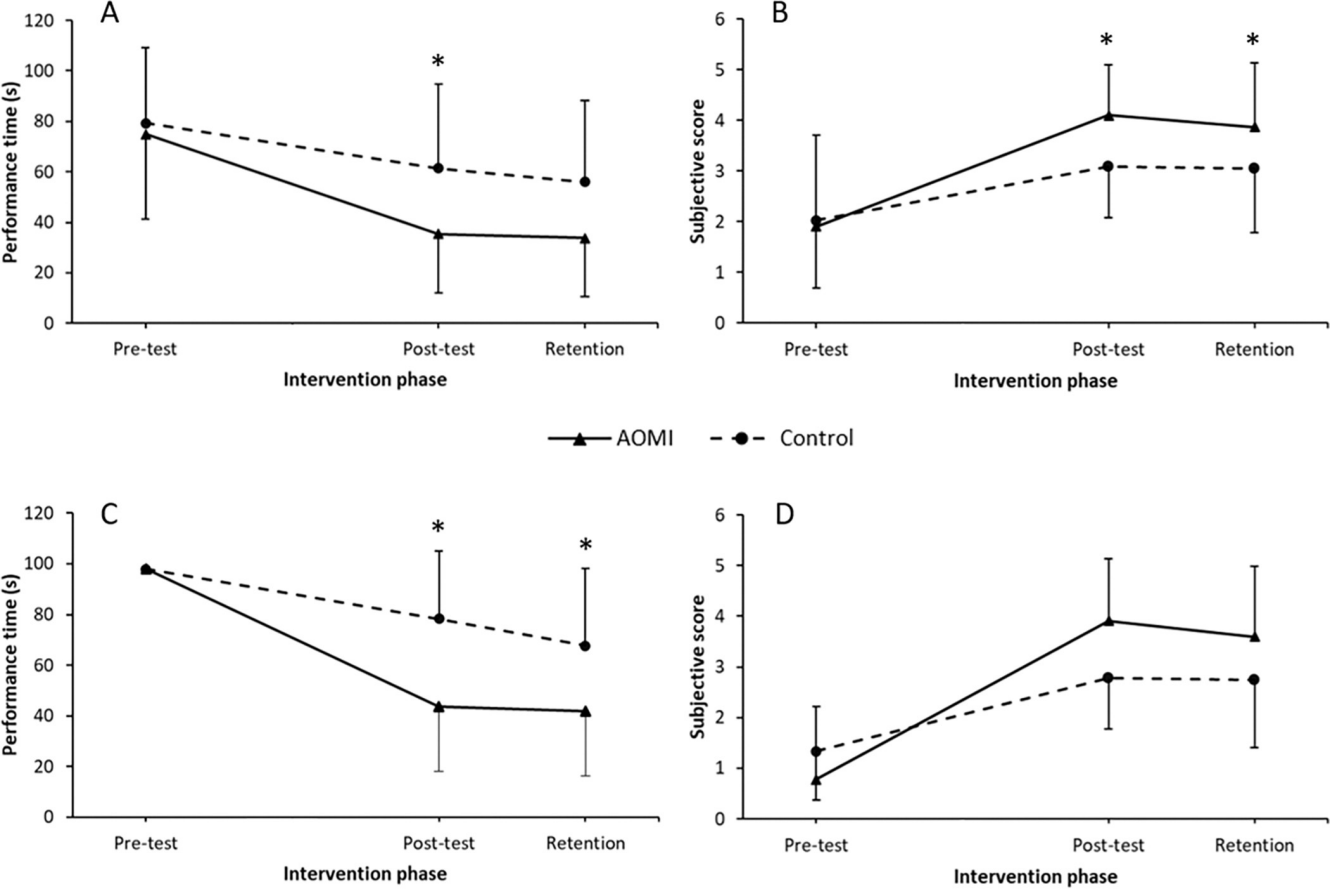
Shoelace tying data. Mean performance time (Panel A) and technique rating (Panel B) data across each test phase (Pre-test, Post-test, Retention) for the combined action observation and motor imagery (AOMI) and control groups for shoelace tying. Mean performance times and technique rating data for children unable to perform shoelace tying at pre-test (n = 9 per group) are presented in Panels C and D, respectfully. Error bars represent standard deviations of group means. * = significant differences between groups.

As expected, all children performed significantly faster at both post-test (*M* = 48.06, *SD* = 31.4, *p* = 0.009) and retention (*M* = 44.53, *SD* = 30.03, *p* = 0.002), compared to pre-test (*M* = 77.12, *SD* = 31.74). There was no significant difference between post-test and retention performance times (*p* = 0.24). Importantly, there was a significant interaction which indicated that at post-test the AOMI group (*M* = 35.51, *SD* = 23.42) performed significantly faster than the control group (*M* = 61.4, *SD* = 33.41, *p* = 0.045). This difference, however, was not retained at the retention phase (*p* = 0.162), despite a visual trend in that direction. See Fig 3A for data profiles.

Interestingly, at pre-test only 10 out of 28 children were capable of performing the shoelace tying task (5 children per group), with a sub-sample of 18 children (9 per group) unable to complete this task successfully. Within this sub-sample, 89% (eight out of nine) of children following the AOMI intervention were able to complete this task successfully by the post-test, while only 44% (four out of nine) of those following the control intervention were able to perform the task after training. An exploratory analysis was conducted including data only from those unable to complete the shoelace tying task successfully at pre-test (See Fig 3 Panel C). This 2 (group) x 3 (time) mixed measures ANOVA revealed a significant group x time interaction, *F*(2,32) = 4.5, *p* = 0.018, $\eta_{p}^{2}$ = 0.22. As shown in Fig 3C, children in the AOMI group performed this task successfully significantly faster at both post-test (*M* = 44.14, *SD* = 24.04) and retention (*M* = 41.24, *SD* = 24.53), compared to the control group (post-test, *M* = 76.99, *SD* = 25.98, *p* = 0.003; retention, *M* = 64.9, *SD* = 35.64, *p* = 0.027).

#### Technique rating

For the ratings of movement technique, all children significantly improved at post-test (*M* = 3.6, *SD* = 1.58, *p* = 0.002) and retention (*M* = 3.48, *SD* = 1.32, *p* = 0.004), compared to pre-test (*M* = 1.97, *SD* = 1.58). Comparison of post-test and retention test performances revealed no difference (*p* = 0.671). A significant interaction was revealed at post-test (*p* = 0.002), with the technique used by the AOMI group (*M* = 4.1, *SD* = 1) found to be significantly better than the technique used by the control group (*M* = 3.09, *SD* = 1.02). This effect was also evident at retention (*p* = 0.011), where the technique of those in the AOMI group (*M* = 3.86, *SD* = 1.25) was significantly better than that of the control group (*M* = 3.06, *SD* = 1.27). See Fig 3B.

Further analysis of participants unable to tie shoelaces at pre-test revealed no significant group differences for movement technique at post-test (*p* = 0.08) or retention (*p* = 0.18), see Fig 3D.

### Cup stacking

#### Performance time

**Fig 4:**
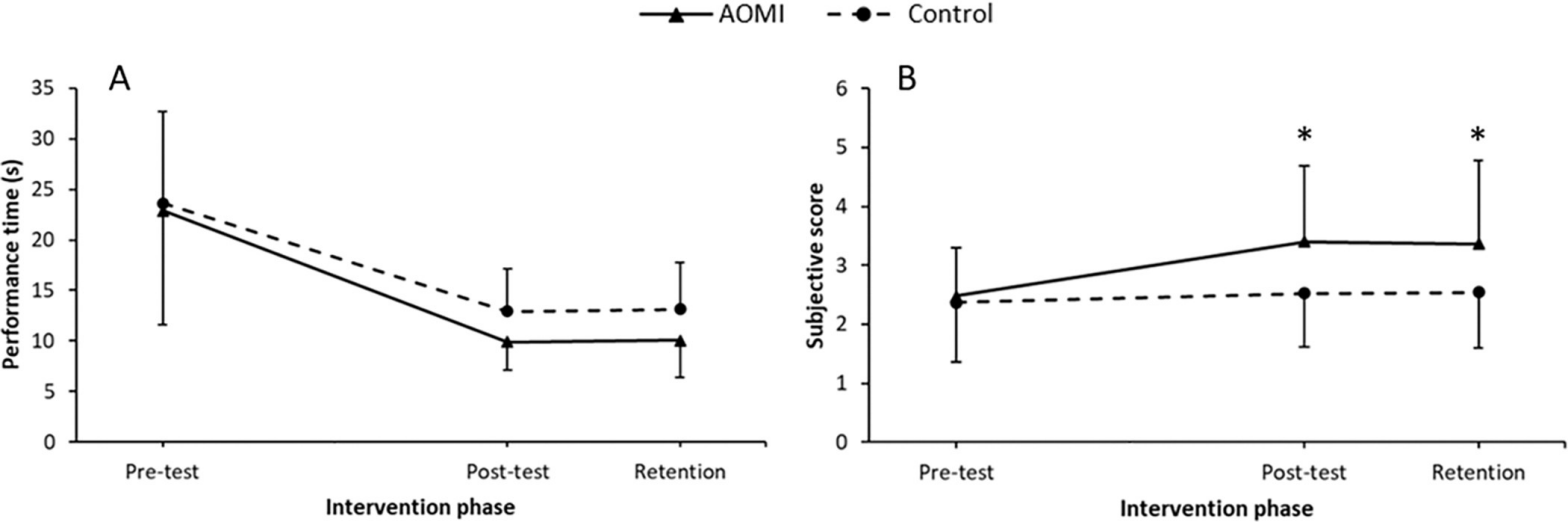
Cup stacking data. Mean performance times (Panel A) and technique ratings (Panel B) data profiles across test phases (Pre-test, Post-test and Retention) for the cup stacking task. Error bars represent standard deviations of group means. * = significant differences between groups.

Children showed significant improvements in performance at both post-test (*M* = 11.43, *SD* = 3.88, *p* < 0.001) and retention (*M* = 11.6, *SD* = 4.43, *p* < 0.001) compared to pre-test (*M* = 23.26, *SD* = 10.24). Post hoc testing revealed no differences between post-test and retention performance times (*p* = 0.947). Furthermore, no group differences were revealed at post-test or retention phases (*ps* > 0.05). See Fig 4A.

#### Technique rating

Analysis of technique ratings showed that all children improved techniques across post-test (*M* = 2.96, *SD* = 0.87, *p* < 0.001) and retention (*M* = 2.96, *SD* = 1.27, *p* < 0.001), when compared to the pre-test (*M* = 2.42, *SD* = 0.91). Comparison of post-test and retention techniques were not significant (*p* = 0.99). As shown in Fig 4B, a significant interaction was revealed at post-test wherein the AOMI group (*M* = 3.4, *SD* = 1.29) had a better technique than the control group (*M* = 2.53, *SD* = 0.91, *p* = 0.008). This group difference was also present at retention (*p* = 0.01), where the technique of the AOMI group (*M* = 3.37, *SD* = 1.41) was significantly better than the control group (*M* = 2.54, *SD* = 0.94).

### Shirt buttoning

#### Performance time

**Fig 5:**
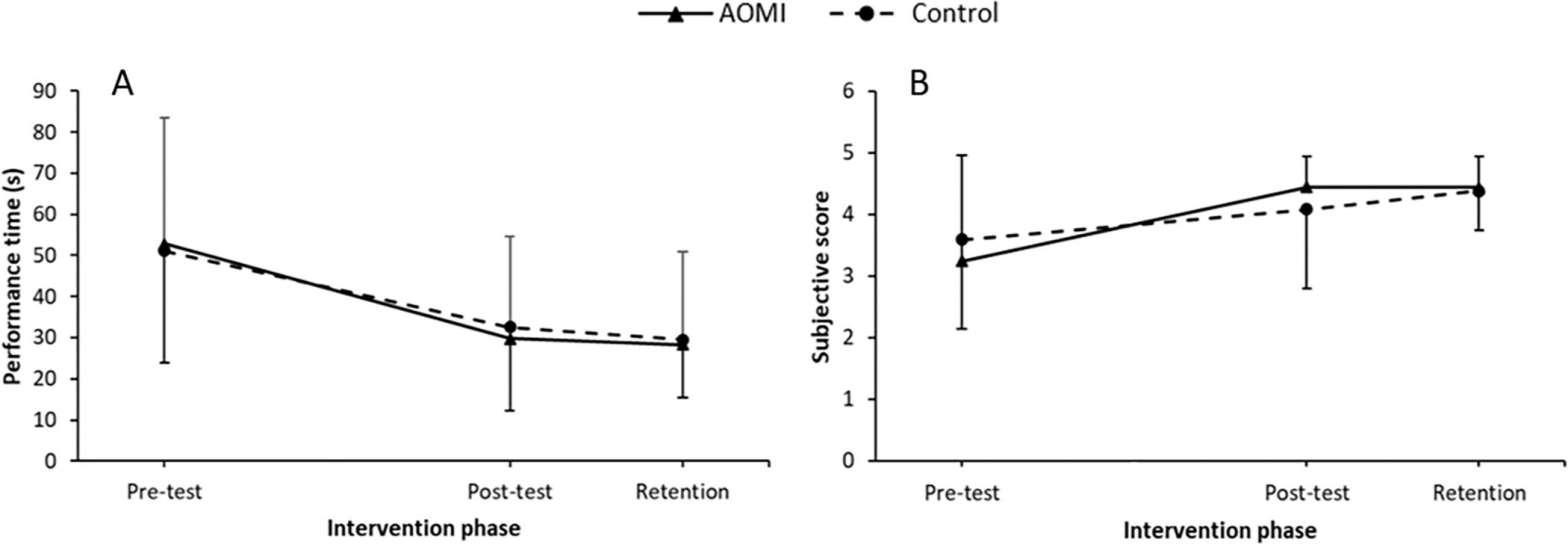
Shirt buttoning data. Mean performance times (Panel A) and technique ratings (Panel B) data profiles across for the combined action observation and kinaesthetic imagery (AOMI) and control group across test phases (Pre-test, Post-test and Retention) for the shirt buttoning task. Error bars represent standard deviations of group means.

All children showed significant improvements at post-test (*M* = 31.18, *SD* = 19.88, *p* = 0.002) and retention (*M* = 28.92, *SD* = 17.47, *p* = 0.001) compared to the pre-test (*M* = 51.96, *SD* = 30.68). As can been seen in the data profiles of Fig 5A, comparison of post-test and retention phases indicated no differences in performance (*p* = 0.906). Furthermore, no differences were identified between groups at any of the test phases (*ps* > 0.05).

#### Technique rating

A significant difference between pre-test (*M* = 3.42, *SD* = 1.58) and post-test (*M* = 4.26, *SD* = 0.99, *p* = 0.02) technique rating scores was identified, where techniques were better at post-test (See Fig 5B). This improvement in technique was retained at the retention phase (*M* = 4.41, *SD* = 0.57, *p* = 0.032). No technique differences were revealed between the post-test and retention test (*p* = 0.972). There were no significant interactions between groups and test phase (*ps* > 0.05).

### Cutlery use

#### Performance time

**Fig 6:**
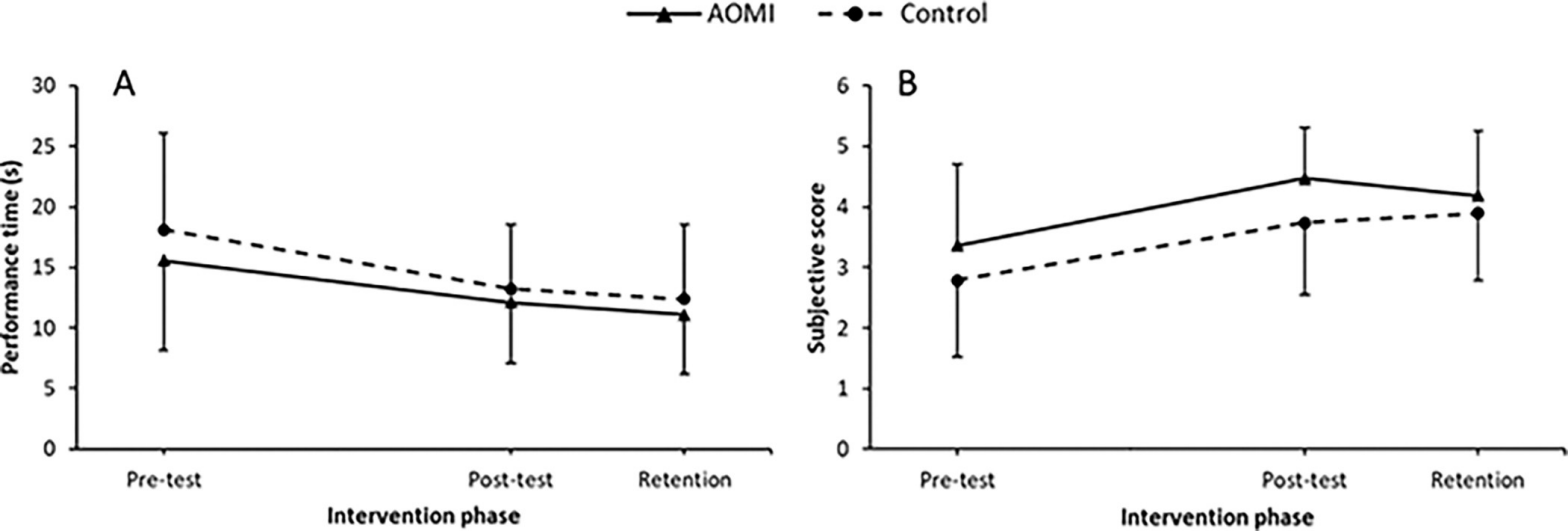
Cutlery use data. Mean performance times (Panel A) and technique ratings (Panel B) data profiles across for the combined action observation and kinaesthetic imagery (AOMI) and control group across test phases (Pre-test, Post-test and Retention) for the cutlery use task. Error bars represent standard deviations of group means.

Children significantly improved their performance times at post-test (*M* = 12.7, *SD* = 5.19, *p* = 0.026) and retention (*M* = 11.76, *SD* = 5.56, *p* = 0.005) compared to the pre-test (*M* = 16.87, *SD* = 7.79). No differences between post-test and retention performances were found (*p* = 0.148) and no significant group differences were revealed across test phases (*ps* > 0.05; see Fig 6A).

#### Technique rating

As shown in Fig 6B, all children improved technique from pre-test (*M* = 3.08, *SD* = 1.33) to post-test (*M* = 4.1, *SD* = 1.09, *p* = 0.004) and pre-test to retention (*M* = 4.04, *SD* = 1.09, *p* = 0.001). No significant difference was found between post-test and retention phases (*p* = 0.71). Comparisons of groups across test phases revealed no significant interactions (*ps* > 0.05).

## Discussion

The purpose of the present study was to investigate the effect of a 4-week AOMI intervention on the performance and learning of four ADLs in children with DCD. Based on previous AOMI research in this population [35, 36], it was hypothesised that AOMI training would facilitate improvements in task completion times and movement techniques compared to the control group across all ADLs. The results provide partial support for this hypothesis. Whilst there was evidence of motor learning in both groups across all four tasks, the expected benefits for the AOMI group relative to the control group were only evident for the shoelace tying and cup stacking tasks but not for the shirt buttoning and cutlery use tasks. For the tasks where beneficial effects of the AOMI intervention were identified, the improved movement techniques were maintained at the retention test which indicates that the AOMI intervention produced motor learning benefits beyond those obtained via physical practice alone. In addition, the secondary analysis comprising only those children who were unable to complete the shoelace tying task at pre-test revealed that the AOMI intervention was more effective in facilitating the learning of this fundamental skill than the control intervention. This indicates that AOMI may be particularly beneficial for aiding the learning of skills not currently within the motor repertoire of children with DCD.

The finding that AOMI improvements only occurred in the shoelace tying and cup stacking tasks may be explained by the relative complexity of the four ADLs. Motor skill complexity refers to the number of different components involved in a skill, with higher complexity skills comprising more individual components [62]. The shoelace tying and cup stacking tasks were arguably of higher complexity than the shirt buttoning and cutlery use tasks. Although the shirt buttoning and cutlery use tasks required the execution of multiple movement components, these tasks essentially required the repetition of the same discrete action (i.e., five button fastens or three knife cuts, respectively), which can only be performed in a limited number of ways. In contrast, both the shoelace tying and cup stacking tasks required the children to execute a sequence comprising multiple different discrete movements, which can potentially be completed using a diverse range of strategies. It is possible, therefore, that in DCD populations AOMI interventions may be particularly effective to support the acquisition of more complex tasks involving multiple components. AOMI, however, appears to be no more beneficial than physical practice for facilitating completion of tasks with fewer movement components or that are more repetitive in nature. This finding is consistent with established effects in the observational learning literature. For example, a meta-analysis of observational learning effects by Ashford et al. [63] demonstrated that observational learning is particularly effective for the acquisition of complex serial skills, compared to discrete or continuous skills. There is also evidence that motor execution of children with DCD is comparable to typically developing children for simple visuomotor tasks, yet their performance declines relative to the typically developing children as task complexity increases [64]. This indicates that children with DCD may require additional support for more complex tasks, and the current findings indicate that AOMI may be a suitable method to provide such support.

There are two, not mutually exclusive, explanations for the beneficial AOMI effects reported in this study. The first explanation relates to the correspondence between the brain regions reportedly hypoactive in children with DCD and those active during AOMI. Motor areas most frequently identified to have supressed activations during movement execution and motor simulation in DCD populations include the inferior frontal gyrus (IFG), precentral gyrus and cerebellum. These areas are important for movement planning, execution and coordination [8, 10]. Although this study did not measure neurophysiological activity, AOMI has been shown to activate these same motor related regions (see [8]). Accordingly, it is plausible that AOMI induces activation across the IFG, precentral gyrus and cerebellum in children with DCD which may contribute to improved motor performance. Such a process has been proposed to be underpinned by long-term potentiation (i.e., Hebbian learning; [29]). An alternative explanation for these improvements is that while using AOMI, the children were provided with the opportunity to pair their MI-generated forward model with an accurate real-time visual display [8, 36]. The cerebellum is believed to generate forward models allowing the prediction of the sensory consequences of movements [65]. In accordance with the prominent IMD hypothesis, this could explain the improved performance and techniques on shoelace tying and cup stacking tasks. AOMI may provide a unique opportunity to update and refine the imagined predicted feedback accordingly during observation [66]. This process, interleaved with physical practice, may have increased the salience of sensory prediction errors during performances leading to attenuation and learning.

Examination of the technique data for the shoelace tying and cup stacking tasks supports the argument that AOMI interventions can contribute to improved imitation ability in children with DCD (see [35] for similar effects). For cup stacking, the pre-test technique scores reflected the initial use of a single-handed strategy by all participants (i.e., scores < 3; see Fig 4). This is unsurprising as children with DCD are known to exhibit poor bimanual coordination [67]. At post-test, however, the mean score of 3.4 for the AOMI group is reflective of these participants adopting a bimanual strategy to complete the task, as modelled in the video, whilst the control participants’ mean score of 2.53 reflects continued use of a single-handed strategy. Similarly for shoelace tying, the mean score of 4.1 in the AOMI group at post-test represents task completion using the modelled technique, compared to 3.09 in the control group which represents task completion with an alternative technique to that modelled (see S2 File for technique rating scales). This indicates that the AOMI intervention was particularly effective in helping the children refine their movement technique by adopting a potentially more efficient movement strategy [68, 69] and thus may prove to be a valuable addition to motor-based therapies. In particular, it has previously been argued that children with DCD may lack the motor problem-solving skills to further develop their motor performance [70], and research indicates that interventions like the Cognitive Orientation to daily Occupational Performance (CO-OP; [71]) that target the development of these skills have been most effective in improving motor skills in DCD. As such, it would be valuable to explore the impact of combining AOMI with CO-OP style interventions–the addition of AOMI may be particularly useful for children who find it more difficult to develop their motor problem-solving skills through CO-OP.

It was hypothesised that the improvements identified in the AOMI group at post-test would be maintained at the retention phase. It is noteworthy, however, that only the improved movement techniques acquired by the AOMI group for shoelace tying and cup stacking remained evident at retention, yet the performance time difference between groups were not retained. These different effects for performance time and movement technique are understandable, however, given that the study employed a relatively long retention period of two weeks during which participants were asked to refrain from any practice of the tasks. Under these circumstances, it is perhaps unsurprising that group differences in performance times may have diminished due to lack of practice, yet importantly the improved movement technique acquired during the intervention period was maintained. This is a crucial finding as the retained group differences for technique provide evidence that the AOMI intervention produced the relatively permanent changes in behaviour indicative of motor learning. This is a novel finding as relatively few previous AOMI studies have used designs with retention tests, including the previous studies with DCD populations [35, 36], but it is consistent with the wider observational learning literature which shows greater benefits for improving movement technique compared to movement outcome [63].

Perhaps the most interesting finding in this study is that AOMI interventions may be particularly effective in supporting children with DCD to learn movement skills that do not currently exist within their motor repertoire. Most participants in this study were able to execute the cup stacking, shirt buttoning and cutlery use ADLs successfully at pre-test, but this was not the case for the shoelace tying task. Only 10 out of the 28 participants in the study could successfully tie their shoelaces at pre-test, with the remaining 18 participants unable to complete this task. Rather than exclude these 18 participants from the study, they were permitted to participate and attempt to learn the skill during the intervention phase. Furthermore, the group randomisation process resulted in nine of these 18 participants being assigned to each group. At the end of the 4-week training period, within this sub-group of participants, eight out of nine (89%) in the AOMI group could tie their laces successfully, yet only four out of nine (44%) in the control group could complete this task. In addition, the statistical analysis confirmed that task performance was significantly better in the AOMI group at post-test and this difference was maintained at retention. Given the inability of these participants to complete this task at pre-test, this represents a true motor learning benefit for AOMI, as opposed to motor refinement in those who could already perform the task [68]. This corroborates previous research showing benefits for AOMI training in individuals with limited or no proficiency in a task [30, 36, 72], and provides important evidence for therapists that AOMI may be particularly beneficial in supporting children with DCD to learn motor skills that do not currently exist in their motor repertoire.

The current study aimed to expand on previous findings showing beneficial effects of acute AOMI training in abstract motor tasks for children DCD (see [34–36]), through a longitudinal investigation of AOMI effects on four fundamental ADLs. In light of the current findings, an appropriate next step would be the development and delivery of a larger scale randomised controlled trial to assess the overall feasibility and effectiveness of AOMI interventions for children with DCD. A particulary helpful development would now be to investigate the feasibility of integrating AOMI as an adjunct to current therapy practices, with feedback from children, families and therapists being used to tailor the intervention content and delivery method. Furthermore, as discussed, it is important to establish the underpinning mechanisms through which AOMI benefits occur [8]. Neurophysiological investigations of AOMI in DCD, therefore, would be a welcome and fruitful addition to the literature to improve the understanding and optimisation of AOMI interventions in this population. Measurement of neurophysiological activity during AOMI use, and pre-post intervention, would be of particular interest in future.

This study adds to the expanding body of literature evidencing benefits for simulation-based interventions in children with DCD [8, 19, 26]. In this instance benefits were found when action observation was *combined* (i.e., synchronised) with motor imagery (AOMI) on complex, bimanual movement tasks. This is the first study to show beneficial effects of home-based AOMI interventions for developing performance on fundamental ADLs in children with DCD over a longitudinal period. While both groups improved their performances, AOMI was more effective in supporting the more complex ADLs, and the greatest benefits were found for children with the lowest proficiency in these tasks. These findings align with recent research reporting benefits for AOMI training in children with DCD [35, 36]. Moreover, the current findings provide task-dependent support for AOMI training as a home-based intervention in children with DCD ranging from 7–12 years old. Accordingly, AOMI may offer a potentially useful intervention approach for therapists to prescribe to aid motor skill acquisition in children with DCD, and the approach may be particularly useful for children who are attempting to learn complex skills or who are currently unable to complete certain skills successfully. Implementation of such an approach could be achieved relatively easily and in a cost-effective manner. For example, therapists could work with parents and train them to administer AOMI interventions tailored to specific complex motor skills with which a child with DCD is struggling or wants to learn, and parents could then integrate the delivery of the intervention into existing family routines.

## Supporting information

S1 File Rating scales created for the Young Scientist’s Diary and adult booklet. These scales allowed children to record their enjoyment and allowed parents to record training dates, times and their child’s motivation and progress.
(DOCX)

S2 File Technique rating scales. These scales were used to measure movement technique for each activity of daily living.
(DOCX)

S3 File Complete results from linear mixed effects models for performance time analyses. (DOCX)
