# Supplementary material for: Combined action observation and motor imagery improves learning of activities of daily living in children with Developmental Coordination Disorder
Source: PLoS One. 2023 May 23;18(5):e0284086. doi: 10.1371/journal.pone.0284086 (PMC10204989; doi:10.1371/journal.pone.0284086)
Supplement: S1 File — These scales allowed children to record their enjoyment and allowed parents to record training dates, times and their child’s motivation and progress. (DOCX) [file pone.0284086.s001.docx]

|  | **How did you feel about your training day?**  **(✓ the one which matches your mood)** | | | | | **Tell us how you feel it went? What was good? What was bad?** | **Did you enjoy the tasks? Were some easy or some hard?** |  |
| --- | --- | --- | --- | --- | --- | --- | --- | --- |
| **Training day 1** | | 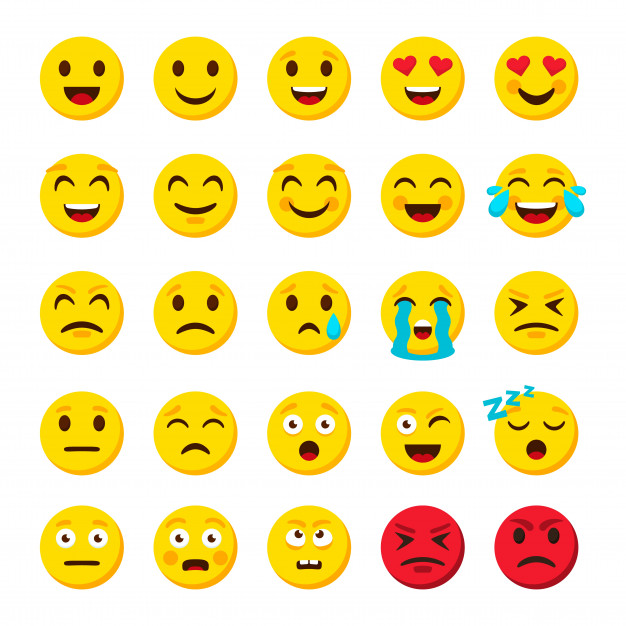 | 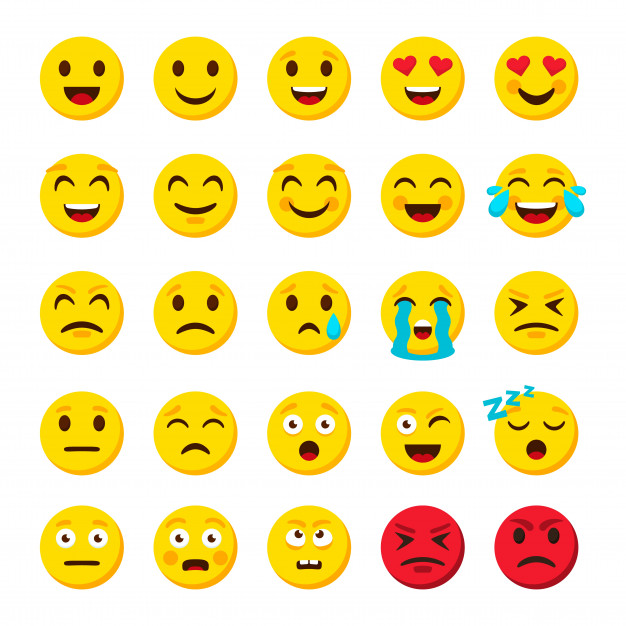 | 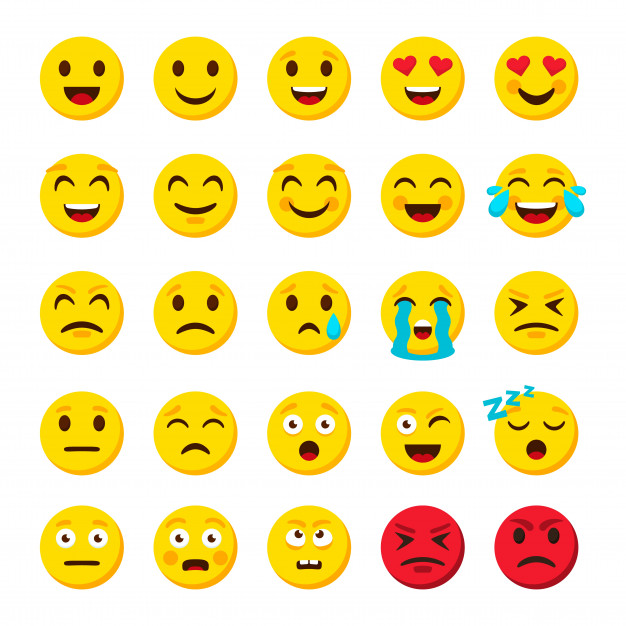 | 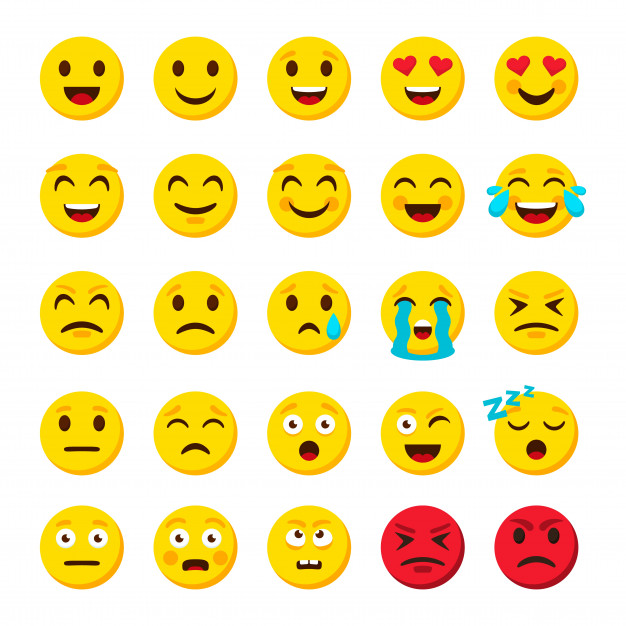 | 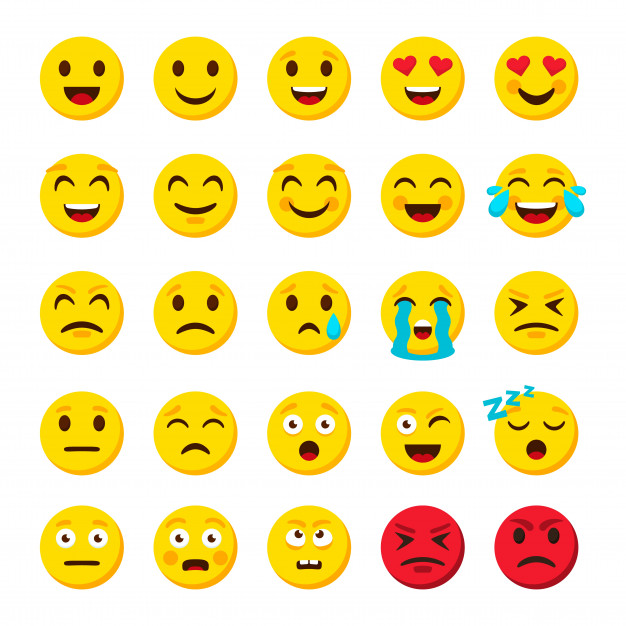 |  |  |
| **Training day 2** | | 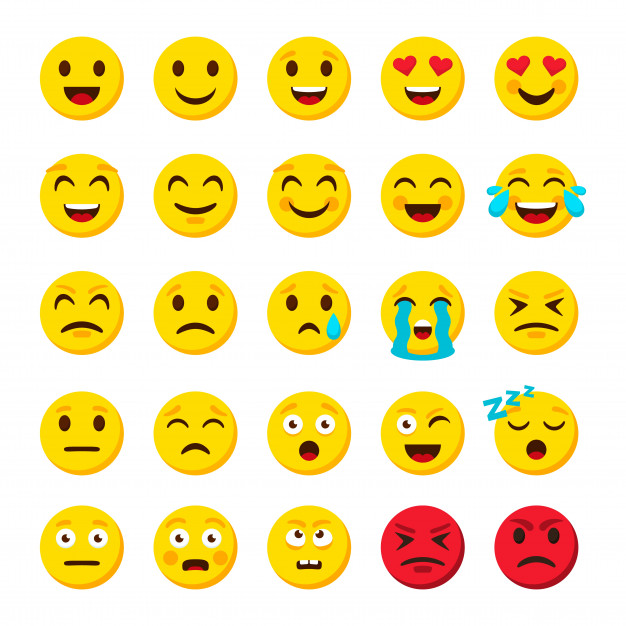 | 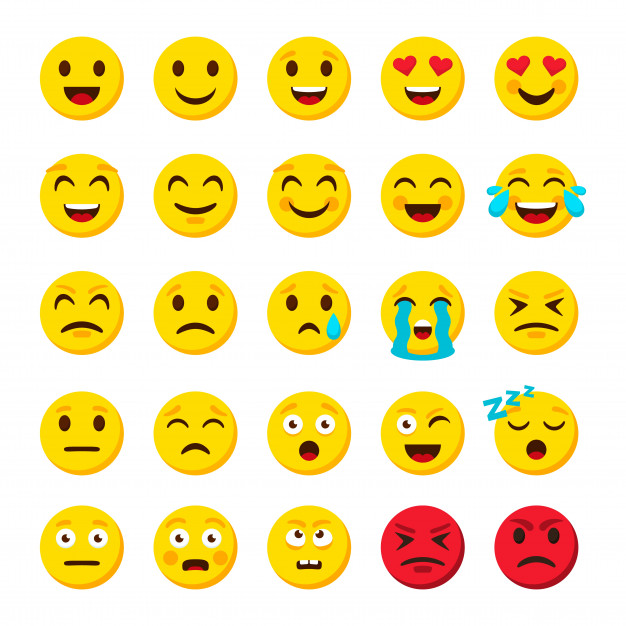 | 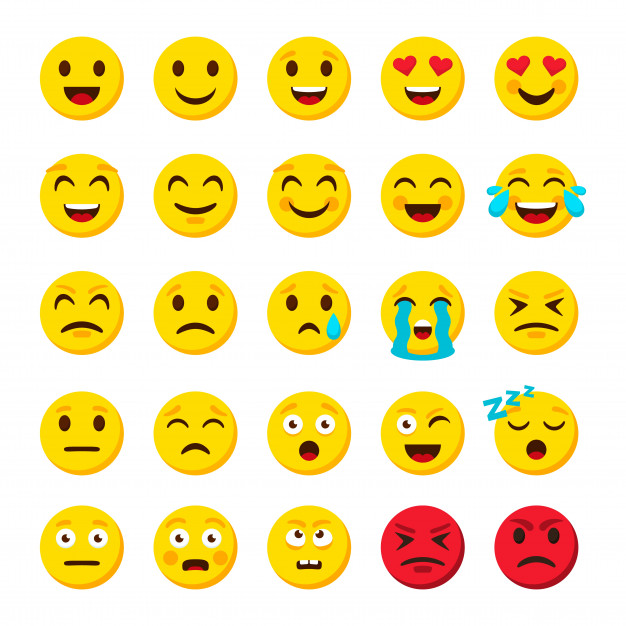 | 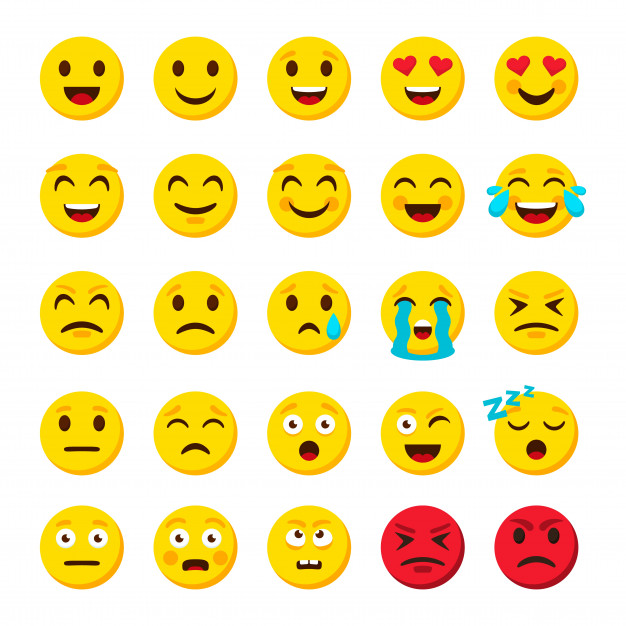 | 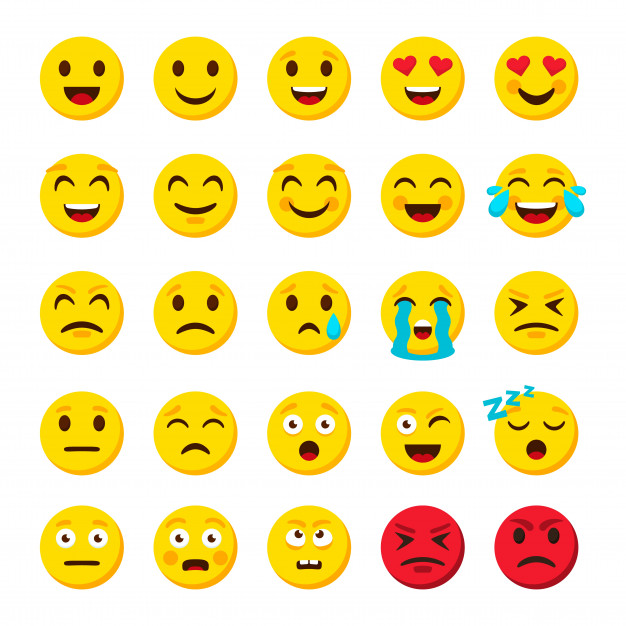 |  |  |
| **Training day 3** | | 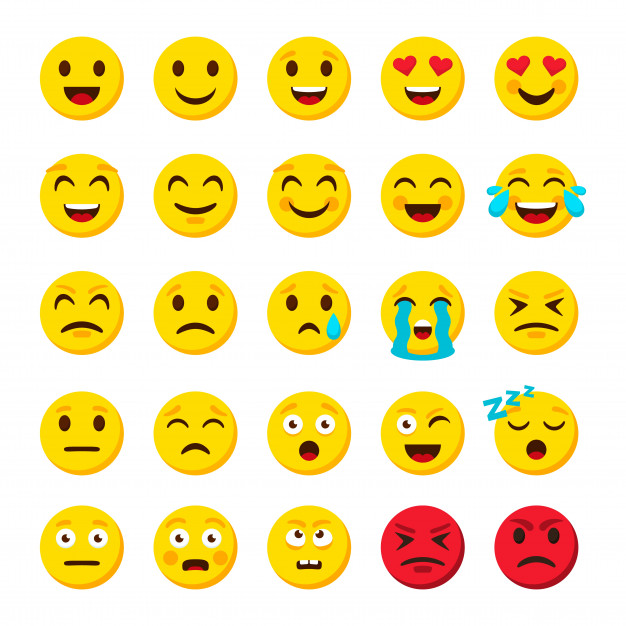 | 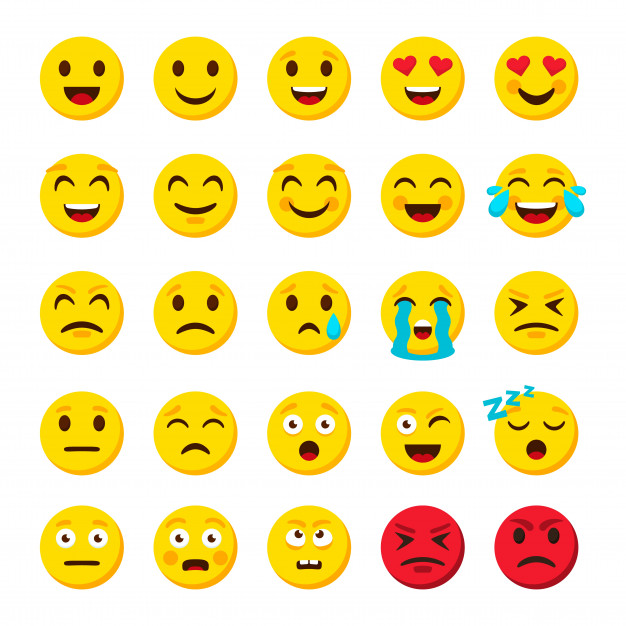 | 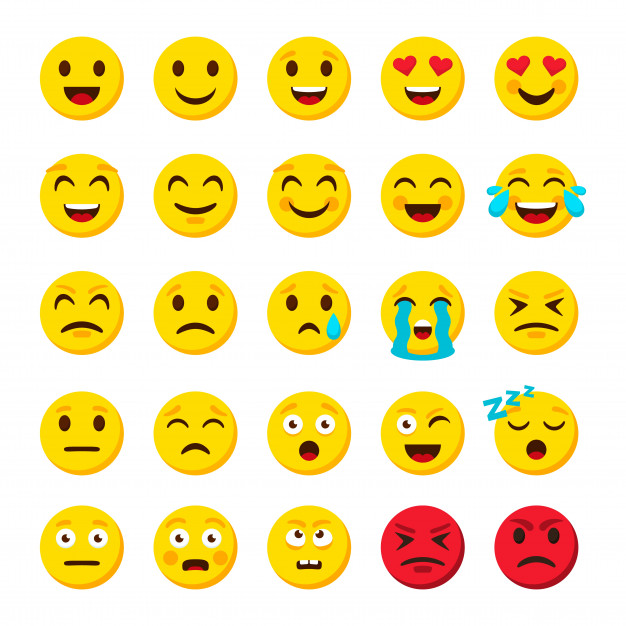 | 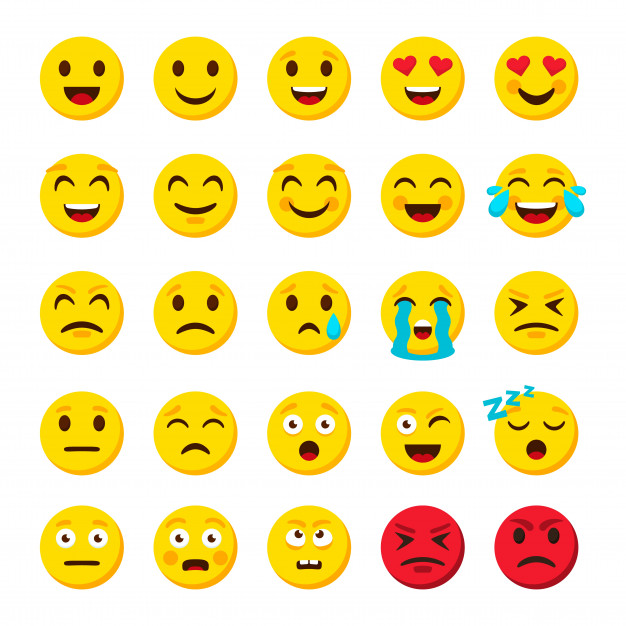 | 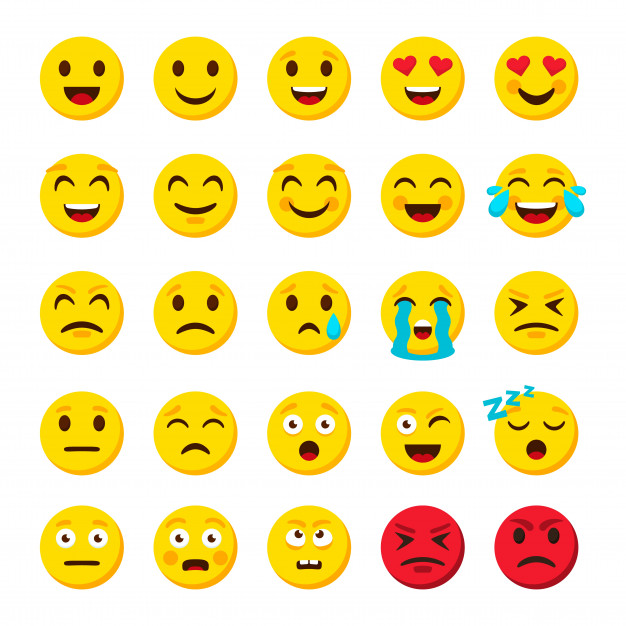 |  |  |
| **Training day 4** | | 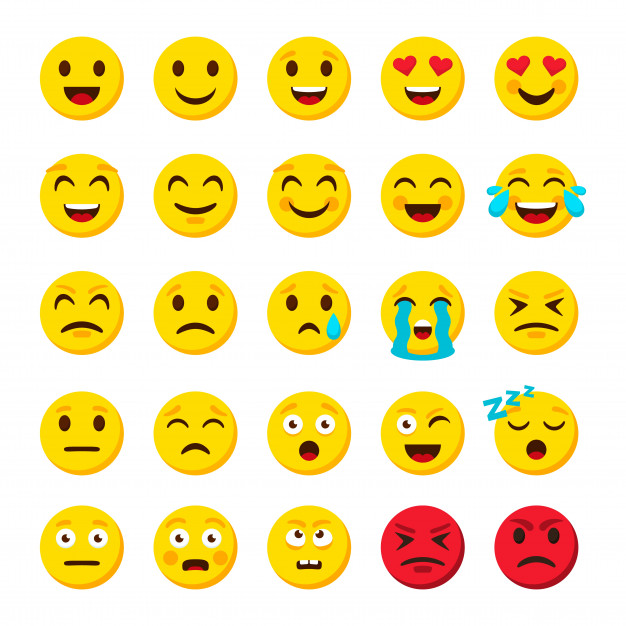 | 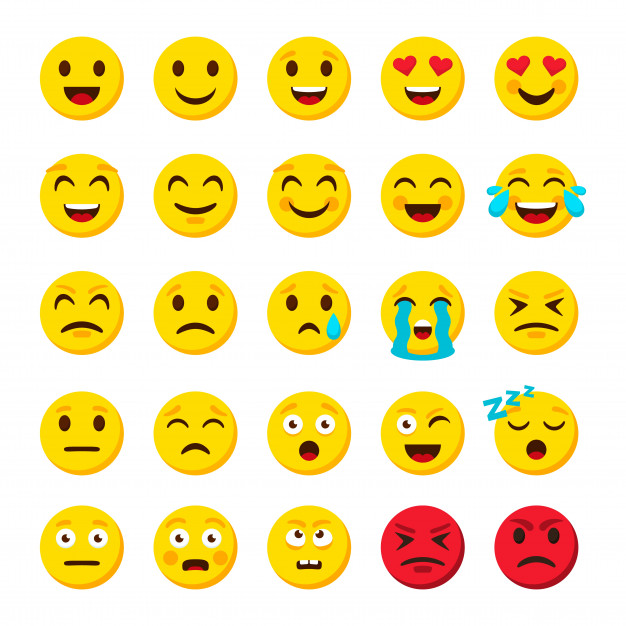 | 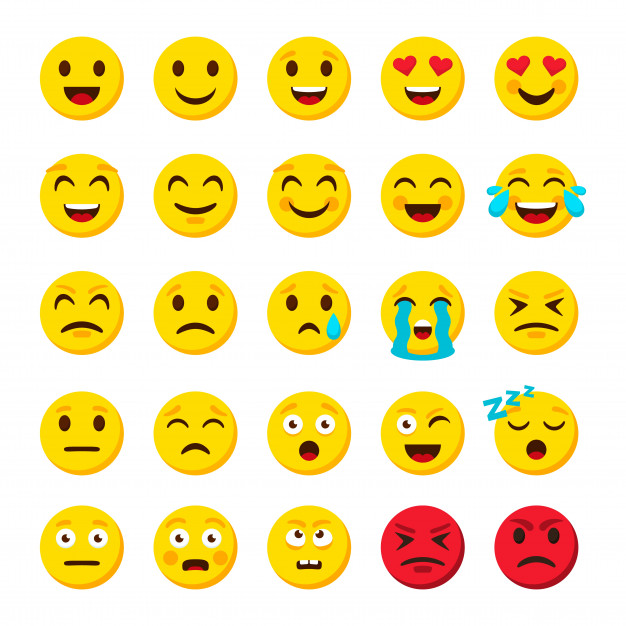 | 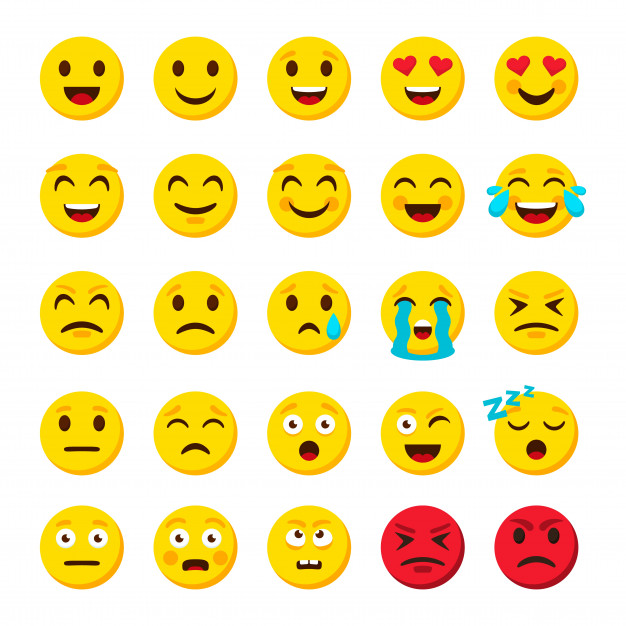 | 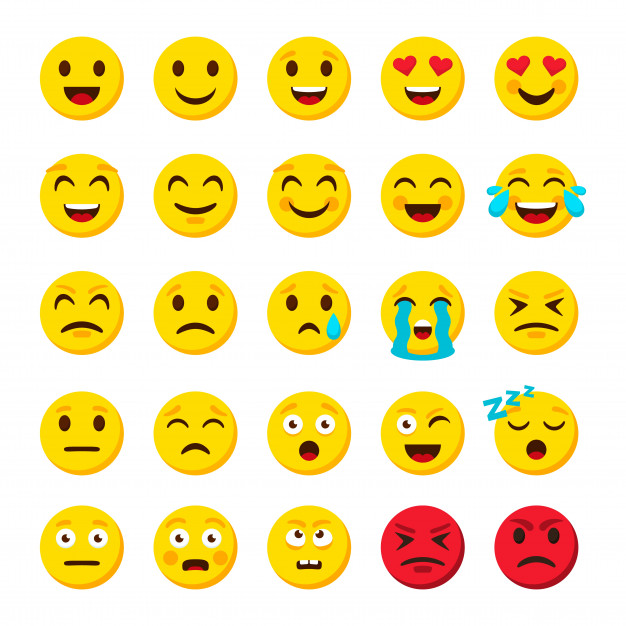 |  |  |

Please work through this booklet during the study. As our Young Scientist this is where you will keep notes for us about what went well and what went less well! Tell us how you felt about each training session and how you feel the training week went.

How do you feel the week went? Do you think your performance on the movement tasks has changed?

………………………………………………………………………………………………………………………………………………………………………………………………………………………………………………………………………………………………………………………………………………………………………………………………………………………………………………………………………………………………………………………………………………………………………………………………………………………………………………………………………………………………………………………………………………………………………………………………………………………………………………………………………………………………………………………………………………………………………………………………………………………………

|  | | **Training task** | | | | **Training session summary** | |
| --- | --- | --- | --- | --- | --- | --- | --- |
| **Training session** | | **Knife and fork** | **Shoelace tying** | **Shirt buttoning** | **Cup stacking** | **Overall motivation for session**  **(1-5)** | **Comments for the session** |
| **Session 1**  Date:……………  Start time:  …………………  Finish time:  ………………… | Motivation rating  (1-5) |  |  |  |  |  |  |
|  | No. of completions |  |  |  |  |  |  |
|  | Comments |  |  |  |  |  |  |
| **Session 2**  Date:……………  Start time:  …………………  Finish time:  ………………… | Motivation rating  (1-5) |  |  |  |  |  |  |
|  | No. of completions |  |  |  |  |  |  |
|  | Comments |  |  |  |  |  |  |
| **Session 3**  Date:……………  Start time:  …………………  Finish time:  ………………… | Motivation rating  (1-5) |  |  |  |  |  |  |
|  | No. of completions |  |  |  |  |  |  |
|  | Comments |  |  |  |  |  |  |
| **Session 4**  Date:……………  Start time:  …………………  Finish time:  ………………… | Motivation rating  (1-5) |  |  |  |  |  |  |
|  | No. of completions |  |  |  |  |  |  |
|  | Comments |  |  |  |  |  |  |
