# Supplementary material for: Combined action observation and motor imagery improves learning of activities of daily living in children with Developmental Coordination Disorder
Source: PLoS One. 2023 May 23;18(5):e0284086. doi: 10.1371/journal.pone.0284086 (PMC10204989; doi:10.1371/journal.pone.0284086)
Supplement: S2 File — These scales were used to measure movement technique for each activity of daily living. (DOCX) [file pone.0284086.s002.docx]

**Subjective rating scales**

**Cup stacking**

| **0** | **1** | **2** | **3** | **4** | **5** |
| --- | --- | --- | --- | --- | --- |
| Task not completed  (if any stack incomplete) | Stacks completed, but cups knocked down | Stacks completed with one hand | Stacks completed using both hands (participant used an alternative technique or had to re-start stack as inappropriate strategy was used) | Stacks completed with close technique to the demonstration | Stacks completed with the demonstrated technique |

Task completion = 3 total stacks completed (3-6-3)

**Shoelace tying**

| **0** | **1** | **2** | **3** | **4** | **5** |
| --- | --- | --- | --- | --- | --- |
| Task not completed (children could not make the first knot) | Participants completed the initial knot  (i.e., over-and-under) | Participants created the first bow but could not continue the task beyond this point | Task completed (lace tied using an alternative technique than demonstrated) | Laces were tied using the demonstrated technique | Laces were tied using the demonstrated technique and laces and bows were balanced |

Task completion = Laces tied using a shoelace bow

**Shirt buttoning**

| **0** | **1** | **2** | **3** | **4** | **5** |
| --- | --- | --- | --- | --- | --- |
| Task not completed | Buttons misaligned | Task partially completed  (2 buttons completed) | Task partially completed  (3-4 buttons complete) | Task completed but grip selection hindered performance (fumbling issues) | The shirt was buttoned with the demonstrated technique, equal time taken for each button |

Task completion = 5 total buttons to be fastened

**Cutlery task**

| **0** | **1** | **2** | **3** | **4** | **5** |
| --- | --- | --- | --- | --- | --- |
| Task not completed | Task partially completed (item not cut into the correct number of pieces) | Task completed but not grasping cutlery with a conventional grip (e.g., inverting fork or fork held backwards) | Task completed but not using appropriate technique (e.g., chopping, ripping or pulling rather than sawing) | Task completed with close technique as demonstrated (hands may have been used to turn plate) | Task completed with the demonstrated technique |

Task completion = Item cut into 4 pieces using 3 cuts
