# Supplementary material for: Combined action observation and motor imagery improves learning of activities of daily living in children with Developmental Coordination Disorder
Source: PLoS One. 2023 May 23;18(5):e0284086. doi: 10.1371/journal.pone.0284086 (PMC10204989; doi:10.1371/journal.pone.0284086)
Supplement: S3 File — (DOCX) [file pone.0284086.s003.docx]

|  | **Shoelace tying** | | | **Cup stacking** | | | **Shirt buttoning** | | | **Cutlery use** | | |
| --- | --- | --- | --- | --- | --- | --- | --- | --- | --- | --- | --- | --- |
| *Predictors* | *Estimates* | *CI* | *p* | *Estimates* | *CI* | *p* | *Estimates* | *CI* | *p* | *Estimates* | *CI* | *p* |
| (Intercept) | 80.42 | 63.53 – 97.32 | **<0.001** | 23.80 | 20.06 – 27.54 | **<0.001** | 52.43 | 36.74 – 68.12 | **<0.001** | 15.84 | 12.41 – 19.26 | **<0.001** |
| Group [AOMI] | -6.93 | -30.81 – 16.96 | 0.570 | -0.90 | -6.18 – 4.38 | 0.738 | -0.75 | -22.94 – 21.44 | 0.947 | 2.18 | -2.66 – 7.02 | 0.378 |
| Time [Post-test] | -18.10 | -31.68 – -4.53 | **0.009** | -10.86 | -14.24 – -7.48 | **<0.001** | -19.01 | -30.76 – -7.26 | **0.002** | -3.82 | -7.18 – -0.46 | **0.026** |
| Time [Retention] | -24.28 | -39.66 – -8.91 | **0.002** | -10.59 | -13.76 – -7.41 | **<0.001** | -20.28 | -32.55 – -8.01 | **0.001** | -4.74 | -8.06 – -1.42 | **0.005** |
| Group [AOMI] * Time [Post-test] | -19.64 | -38.79 – -0.48 | **0.045** | -2.12 | -6.90 – 2.65 | 0.383 | -2.73 | -19.34 – 13.89 | 0.748 | -0.95 | -5.71 – 3.80 | 0.694 |
| Group [AOMI] * Time [Retention] | -15.50 | -37.23 – 6.23 | 0.162 | -2.26 | -6.74 – 2.23 | 0.324 | -2.71 | -20.05 – 14.64 | 0.760 | -0.90 | -5.60 – 3.80 | 0.707 |
| **Random Effects** | **Shoelace tying** | |  | **Cup stacking** | |  | **Shirt buttoning** | |  | **Cutlery use** | |  |
| σ^2^ | 51.30 |  |  | 25.72 |  |  | 59.35 |  |  | 13.00 |  |  |
| τ_00_ _Participant_ | 1027.45 |  |  | 45.52 |  |  | 882.43 |  |  | 39.88 |  |  |
| τ_11_ _Participant.TimeWeek2_ | 644.68 |  |  | 31.13 |  |  | 474.96 |  |  | 35.66 |  |  |
| τ_11_ _Participant.TimeWeek3_ | 836.34 |  |  | 26.01 |  |  | 518.92 |  |  | 34.74 |  |  |
| ρ_01_ | -0.56 |  |  | -0.93 |  |  | -0.78 |  |  | -0.74 |  |  |
|  | -0.60 |  |  | -0.90 |  |  | -0.80 |  |  | -0.70 |  |  |
| ICC | 0.94 |  |  | 0.44 |  |  | 0.90 |  |  | 0.68 |  |  |
| N _Participant_ | 28 |  |  | 28 |  |  | 28 |  |  | 28 |  |  |
| Observations | 391 |  |  | 414 |  |  | 377 |  |  | 406 |  |  |
| Marginal R^2^ / Conditional R^2^ | 0.259 / 0.958 |  |  | 0.415 / 0.674 |  |  | 0.150 / 0.911 |  |  | 0.125 / 0.717 |  |  |
